# Making things happen: How employees’ paradox mindset influences innovative performance

**DOI:** 10.3389/fpsyg.2022.1009209

**Published:** 2022-12-12

**Authors:** Yanjun Liu, Hui Zhang

**Affiliations:** ^1^School of Economics and Management, North China University of Technology, Beijing, China; ^2^School of Sociology, Huazhong University of Science and Technology, Wuhan, China

**Keywords:** paradox mindset, role breadth self-efficacy, individual ambidexterity, innovative performance, social cognitive theory

## Abstract

Individual innovation involves many contradicted behavioral options such as creative vs. habitual actions and explorative vs. exploitative activities. However, the agentic nature of innovative behaviors has been widely ignored, and we know less about what factors lead individuals to approach and balance the contradictions caused by competing demands and intentionally engage in innovative behaviors. Integrating social cognitive theory and innovation paradox, we propose a chain-mediating model to explain how employees with a paradox mindset realize the creative benefits through their innovative endeavors, considering role breadth self-efficacy (RBSE) and individual ambidexterity as two mediators. Using data collected from 480 employees paired with 100 supervisors at 3-time points, the results show that RBSE and individual ambidexterity play a mediating role, respectively, even though they sequentially play a chain-mediating role between employees’ paradox mindset and innovative performance. Individuals who hold a paradox mindset are more likely to perceive high capability beliefs in successfully undertaking expanded roles, promoting behavioral tendencies to switch between exploration and exploitation, and in turn encouraging employees to undertake more innovative behaviors. Finally, we discuss the theoretical and practical implications for promoting employees’ innovative performance from an agentic perspective. Employees with a paradox mindset can make creative things happen by managing the tensions between exploration and exploitation proactively. Thus, organizations may try to enhance employees’ proactive motivation states and behavioral capability to encourage individual innovation.

## Introduction

Employees’ innovation is the key to maintaining business success in modern organizations, which rely on such individual innovation to gain a competitive advantage not only for the change needed for long-term viability but also for incremental improvement of the processes and procedures (Shalley et al., 2015; Knippenberg and Hirst, 2020). Effective innovation emerges from the development and implementation of novel and potentially useful outcomes, including processes, products, practices, and solutions to problems in the workplace (Chen et al., 2013). However, the business demands on innovation are not only simply limited to selecting the employees to devote to research and development but also more broadly include individuals tackling non-routine job challenges (Hirst et al., 2011). Furthermore, innovative and habitual actions usually are regarded as competing for behavioral options (Ford, 1996), which inspires scholars to recognize innovation as paradoxical and explore new strategies for managing innovative tensions (Miron-Spektor et al., 2011a; Miron-Spektor and Erez, 2017).

Considering the paradoxical nature of innovation, scholars pay more and more attention to examining the factors influencing one’s innovative performance at the workplace through a paradox lens (Miron-Spektor and Erez, 2017). Recently, a paradox mindset, as an essential dispositional construct, has been arousing some scholars’ interests and has been studied gradually. A paradox mindset refers to “the extent to which one is accepting of and energized by tensions” (Miron-Spektor et al., 2018, p. 26). They also argue that individuals who carry a paradox mindset tend to value, accept, and feel energized by tensions, which can help the employees to improve in-role job performance and innovation. Liu et al. (2020) also find that a paradox mindset can also stimulate individuals to produce innovative outputs through thriving at work from a motivational perspective. In addition, some studies began to expand the outcomes that a paradox mindset is related to, such as work engagement (Yin, 2021) and work-family conflict (Chen et al., 2020). Although scholars explore the positive effect of a paradox mindset, we only know little about how the paradox mindset motivates individuals to promote their innovative performance.

To engage in innovative actions, individuals should hold a strong sense of agency, which is described as a desire to intentionally make things happen by means of their own actions (Ng and Lucianetti, 2016). Grounded in the human agentic perspective, self-efficacy is recognized that a person possessing beliefs about their capability to perform particular tasks (Gist and Mitchell, 1992). The social cognitive theory suggested that one’s self-efficacy determines behavioral intensity when the domains of those beliefs are consistent with the type of actions in question (Bandura, 2012). Although studies suggest that creative self-efficacy is positively related to employees’ innovation (Tierney and Farmer, 2002; Gong et al., 2009), it cannot promote individual innovation alone (Ng and Lucianetti, 2016). Therefore, employees tend to choose habitual and familiar behavioral options rather than creative actions based on relative certainty and ease as well as their past success (Ford, 1996), thus, we focus on exploring whether other types of self-efficacy also had a positive influence on individual innovation.

Role breadth self-efficacy (RBSE) is referred to people’s judgment about their confidence that they are capable of “carrying out a broader and more proactive role beyond traditional prescribed technical requirements” (Parker, 1998, p. 835). As a type of “can do” motivational state, RBSE can encourage individuals to engage in innovative behaviors (Rodrigues and Rebelo, 2021). Given that employees with a paradox mindset are typically energized to recognize and embrace contradictions between habitual and creative actions, they are more likely to undertake a broader role and generate innovative benefits eventually. Therefore, we propose that RBSE may be a potential explanatory mechanism between employees’ paradox mindset and innovative performance.

**FIGURE 1:**
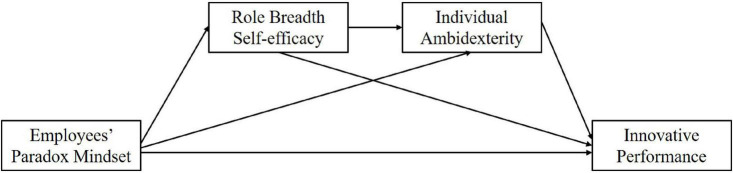
Theoretical model.

Based on Ford’s (1996) model of individual creative action, actions stem from the joint influence of sensemaking, motivation, knowledge, and ability. Although self-efficacy is a key motivational component in this model, knowledge and ability also have a potential influence on individual innovation. Thus, we attend to another possible mediated variable individual ambidexterity, which refers to an individual’s behavioral capacity to engage in and alternate between explorative and exploitative tasks in their work roles (Mom et al., 2009; Kauppila and Tempelaar, 2016). Compared to employees who just focus on either exploration or exploitation, those who consider both are more creative because they are energized by the integration of paradoxical demands, which prevents them from taking refuge in their habitual thoughts (Miron-Spektor et al., 2011b; Leung et al., 2018). Therefore, we would like to explore psychological mechanisms that link employees’ paradox mindset and individual innovation from an agentic perspective. As shown in our theoretical model (Figure 1), both RBSE and individual ambidexterity, respectively, play the mediating roles between employees’ paradox mindset and innovative performance. Furthermore, they also constitute a chain-mediating path that can explain how individuals with a paradox mindset make innovative things happen.

The present study aims to make three contributions to understanding how employees’ paradox mindset influences their innovative performance. First, from an agentic perspective, we draw on the tenets of social cognitive theory (Bandura, 2012) to theorize RBSE as the potential mediating path between a paradox mindset and innovative performance beyond the mediating role of thriving at work between them (Liu et al., 2020). Second, we contribute to the individual ambidexterity literature by identifying a paradox mindset as an antecedent of individual ambidexterity. Scholars have been calling for examining individual ambidexterity through a paradox lens (Papachroni and Heracleous, 2020). This study responds to it by building the relationship between a paradox mindset and ambidexterity at the individual level. Third, we provide empirical evidence that how capability beliefs and behavioral abilities in combination can influence individual innovation by exploring the chain-mediating role of RBSE and individual ambidexterity between employees’ paradox mindset and innovative performance.

## Theoretical background and hypothesis development

### Managing innovation paradoxes through a paradox mindset

A paradox denotes persistent contradictions between interwoven elements that seem logical independently but inconsistent when juxtaposed (Smith and Lewis, 2011; Schad et al., 2016). Creative ideas involve “both-and” elements, such as novel and useful, which require individuals to be both learning-orientated and performance-orientated (Miron-Spektor and Beenen, 2015), passionate and disciplined (Andriopoulos and Lewis, 2009), and flexible and persistent (Baas et al., 2013). Thus, innovation is paradoxical inherently, including contradictory and interdependent thoughts, perspectives, processes, and outcomes (Miron-Spektor and Erez, 2017). Furthermore, innovative behaviors do not tend to occur unless they are desired relatively more expected than familiar behaviors and this contradictory aspect of individuals’ behavioral intentions has been ignored (Ford, 1996). Therefore, it is necessary to investigate how employees manage such paradoxical tensions to favor individual innovation in the workplace.

The influence of tensions depends on an individual’s approach to paradox, and specifically, individuals who regard tensions as paradoxes rather than dilemmas are more likely to embrace opposing elements and integrate contradictory perspectives (Smith and Lewis, 2011). Paradoxical frames are defined as mental templates providing a lens to understand a situation to recognize and accept contradictions, thus enabling individuals to embrace and feel comfortable with persistent inconsistences rather than eliminating them (Smith and Tushman, 2005, p. 523; Miron-Spektor et al., 2011b). A paradox mindset can improve an individual’s tendency to confront rather than avoid contradictions. Individuals with a paradox mindset who tend to embrace contradictions and feel energized by tensions are more innovative than those who lack such a mindset (Miron-Spektor et al., 2018). Adopting a paradox mindset encourages the cognitive juxtaposition of inconsistent elements and contributes to integrating opposing task elements to generate new solutions (Miron-Spektor et al., 2011b). In addition, Liu et al. (2020) demonstrate that employees’ paradox mindset is positively related to innovative work behavior. Based on the above analysis, we propose the following hypothesis :

***Hypothesis 1:** Employees’ paradox mindset is positively related to innovative performance.*

### Paradox mindset and role breadth self-efficacy

According to Bandura (2006), an agentic individual is more likely to intentionally make things happen through his or her actions. Individuals with a paradox mindset who tend to confront contradictory demands proactively are more likely to attend to both routine and creative work (Miron-Spektor et al., 2018). This means that they are self-motivated to achieve desired outcomes based on their personal agency. Human agency is the capability of an individual to take actions through self-perception of abilities, planning, framework reconstruction, and evaluation of goals achieved in the social environment (Bandura, 2018). The social cognitive theory argues that self-efficacy is the key to determining whether an actor can successfully influence their behaviors in his or her own way (Bandura, 2006).

There are four principal sources of information on which people base determining their self-efficacy beliefs: enactive mastery, vicarious experiences, verbal persuasion, and emotional arousal (Bandura, 1982; Gist and Mitchell, 1992). We mainly attend to the individual’s physiological states because a series of studies on desensitization show that an individual’s psychological state is one of the important factors changing self-efficacy and diminishing negative emotional arousal that can reduce avoidance behaviors (Bandura, 1977). They also argue that task situations may cause changes in psychological state, which leads to changes in individuals’ judgment of their own ability accordingly. Therefore, we propose that employees who are primed with a paradox mindset may elicit energized feelings (Miron-Spektor et al., 2018; Liu et al., 2020), as a kind of emotional information source, influencing their self-efficacy beliefs.

Compared with general self-efficacy beliefs, RBSE is defined as individuals’ perceived ability to undertake a broader role, involving a variety of interpersonal, proactive, and integrative tasks, beyond prescribed technical work activities (Parker, 1998; Parker et al., 2006). RBSE is considered as a malleable motivational state (Parker et al., 2010). We propose that a paradox mindset as a trait-like factor that activates the perceptions of employees’ own capabilities. First, employees with a paradox mindset are competent to confront tensions produced by contradicted elements (Miron-Spektor et al., 2018). Because a paradox mindset can increase employees’ integrative complexity (Tadmor et al., 2012), promoting their cognitive flexibility and also increasing their willingness and capacity to tolerate and integrate different perspectives (Miron-Spektor et al., 2011b), thus enhancing the beliefs that they can undertake broader roles. Second, employees who accept and value tensions are more likely to feel energized to respond to tensions (Miron-Spektor et al., 2018) and increase the overall resources for them to engage in specific work, which is conducive to improving their competence to perform broader tasks. Third, a paradox mindset may enhance employees’ intrinsic motivation and thriving at work (Liu et al., 2020), which may encourage employees proactively to engage in more than in-role work behaviors (Zeng et al., 2020; Alikaj et al., 2021). Therefore, we suggest that employees’ paradox mindset can promote their RBSE, and we propose the following hypothesis:

***Hypothesis 2:** Employees’ paradox mindset is positively related to role breadth self-efficacy.*

### The mediating effect of role breadth self-efficacy

Innovative behaviors often involve challenging the status quo and introducing contradictory issues in organizations, as well as coping with possible resistance and risk of failure (Hsu and Chen, 2017; Ouyang et al., 2019). Innovation refers to the intentional generation, promotion, and application of new ideas (Janssen, 2004). Ng and Lucianetti (2016) argue that innovative actions can be regarded as a type of agentic behavior. Given that social cognitive theory approaches the cognitive beliefs about agentic behavior (Bandura, 2006), it provides an appropriate theoretical perspective to examine innovative behaviors. Previous studies have shown that self-efficacy beliefs play an important role in enhancing employees’ intention to engage in innovative and changed actions (Sonnentag and Spychala, 2012; Ng and Lucianetti, 2016).

Role breadth self-efficacy is the central mechanism of individuals’ motivation, which determines their emotional and behavioral processes (Schaubroeck et al., 2017). First, high RBSE is supposed as promoting individuals’ perceptions of job control and the possibility of success of their own initiatives, such as bringing improvement and change in the organization (Parker and Collins, 2010). When employees consider that creative ideas can bring benefits to the organization, a high level of RBSE motivates them to carry out innovative activities. Second, high RBSE can help employees to expand their roles, along with improving their resilience, self-confidence, and challenging spirit (Parker et al., 2010). Meanwhile, they are proactive to perform extra tasks beyond completing in-role job performance and also will have the courage to engage in more innovative behaviors. Therefore, in accordance with Chen et al. (2013), we consider that RBSE acts as a key cognitive-motivational state to generate a positive influence on individual innovative performance.

When confronted with an innovation paradox, individuals with a high paradox mindset are energized by tensions from engaging in both habitual and creative actions, which makes this emotional arousal enhances their RBSE. Furthermore, RBSE can play an instrumental role between individuals’ personalities and innovative performance beyond the criterion of proactive behaviors (Chen et al., 2013). Consequently, we propose that individuals with a paradox mindset experience higher RBSE, which denotes one’s confidence in the capability to generate and implement new ideas. Hence, these individuals are motivated to engage in innovative behaviors. Based on this analysis, we hypothesize the following:

***Hypothesis 3:** Role breadth self-efficacy mediates the relationship between employees’ paradox mindset and innovative performance.*

### Paradox mindset and individual ambidexterity

In most contemporary organizations, employees are required to perform their job responsibilities in order to meet prescribed work requirements (Zhang and Bartol, 2010). In addition, they are encouraged to be innovative to put forward and implement new ideas (Amabile et al., 1996). In the management literature, ambidexterity refers to an organization’s ability to pursue explorative and exploitive activities simultaneously (Gupta et al., 2006). At the individual level, exploration is referred to individuals deviating from routines, searching for new or alternative ways to accomplish a task, and not relying on their existing knowledge, while exploitation is referred to individuals performing tasks relying on previous experiences and rules, improving well-learned actions incrementally (Kauppila and Tempelaar, 2016; Rosing and Zacher, 2017).

Given that individual ambidexterity is conceptualized as the combination of distinct dimensions of exploration and exploitation (Mom et al., 2009), it can be enhanced by the factors increasing exploration or exploitation. However, the premise is that the increase of one is not at the cost of decreasing the other (Kauppila and Tempelaar, 2016). Exploration and exploitation are not only simply different kinds of organizational behavior but also complementary and mutually enabling between each other (Holmqvist, 2004; Farjoun, 2010). Separating exploration and exploitation at the individual level may lead to tensions and contradictions. Bledow et al. (2009) argue that the tensions between these activities can be addressed within the same subsystem (e.g., individuals). Individuals who have a paradox mindset can cope with the challenges that are related to the integration of contradictory demands and conflicting agendas (Leung et al., 2018). Therefore, we propose that individuals with a paradox mindset can confront the “exploration-exploitation” paradoxical situation and embrace the tensions between them, in turn promoting individual ambidexterity.

On the one hand, employees carry out both explorative and exploitative activities relying on their intangible resources, such as limited time and knowledge, which leads to the competitive relationship between exploration and exploitation (Martin et al., 2019). Drawing on the tenets of a dynamic equilibrium model of organizing (Smith and Lewis, 2011), environmental factors involving plurality, change, and scarcity can make latent tensions become salient. Specifically, scarcity can be regarded as a limitation on resources, which leads individuals to experience the inconsistent and contradictory nature of the tensions. Employees with a paradox mindset would leverage salient tensions to enhance both in-role job performance and innovation (Miron-Spektor et al., 2018), in turn promoting individual ambidexterity.

On the other hand, a paradox mindset can enhance individuals’ cognitive flexibility and integrative complex thinking (Leung et al., 2018). Furthermore, the sense of energy produced from tensions also can help them to switch between exploration and exploitation (Smith and Tushman, 2005). In contrast, those employees with a low paradox mindset tend to focus on how to eliminate the tensions caused by opposing elements (Miron-Spektor et al., 2011b), which are not conducive for them to engage in both explorative and exploitative activities simultaneously, showing the low level of ambidextrous behaviors. Based on the above analysis, we propose the following hypothesis:

***Hypothesis 4:** Employees’ paradox mindset is positively related to individual ambidexterity.*

### The mediating effect of individual ambidexterity

Tensions, paradox, and contradiction are inherent characteristics of innovation (Lewis et al., 2002; Bledow et al., 2009). At the individual level, it is easy to elicit tensions by engaging in both explorative and exploitative activities at the same time (Martin et al., 2019). Since exploration often leads to failure, individuals need to search for alternative ideas constantly. However, efficiency and reliability are not taken into account when employees are devoted to explorative activities. Whereas exploitation often leads to success, employees who are devoted to exploitative activities all the time may crowd out their needs for broad search and risk-taking capacity (Gupta et al., 2006). Rosing and Zacher (2017) argue that the value of individual ambidexterity to creativity lies in the integration of contradictory demands and paradoxical tensions between explorative and exploitative activities. Studies suggest that ambidextrous leaders can also perform multiple roles to engage in different work activities (Mom et al., 2009, 2015). On the one hand, too much exploration may lead to confusion; on the other hand, too much exploitation may lead to the rigidity (Rosing and Zacher, 2017). In another words, opposing behavioral strategies should be integrated in order to curb the negative impact of each strategy, thus improving innovative performance (Gebert et al., 2010; Pertusa-Ortega et al., 2021). Given that individuals are the smallest behavioral carriers, exploration and exploitation cannot be strictly separated (Birkinshaw and Gupta, 2013). Therefore, it is necessary to integrate explorative and exploitative activities to achieve individual ambidexterity.

Studies on organizational ambidexterity show that the combination of exploration and exploitation has a significantly positive influence on innovation (Gibson and Birkinshaw, 2004; Zacher and Rosing, 2015). We propose that the integration of these two activities at the individual level will also positively predict innovation, that is, both employees’ exploration and exploitation are high, which will promote innovative performance. First, a high level of exploitative activities can promote the positive influences of high explorative activities on innovative performance (Zacher et al., 2016). When employees engage in complying with norms and achieving work goals, they are more likely to transform previous explorative activities into valuable products or services (Rosing and Zacher, 2017). Furthermore, they also perceive themselves as more innovative because exploration and exploitation are thought to reinforce each other mutually (Caniëls and Veld, 2019). Therefore, the integration of exploration and exploitation denotes high ambidexterity that can boost their innovative performance.

Second, when either exploration or exploitation, or both explorative and exploitative behaviors are low, either situation can lead to low innovative performance (Zacher et al., 2016). Specifically, when employees carry out high explorative behaviors and low exploitative behaviors, they perceive them as creative but cannot implement new and useful ideas effectively (Bledow et al., 2009). Thus, they are less likely to achieve high innovative performance. Whereas employees engage in more exploitative activities and less explorative activities, new and useful ideas cannot be generated, so it is inconsistent with the connotation of innovation (Zacher et al., 2016). Finally, when employees engage in both low explorative and exploitative activities, they are not able to introduce and implement new and useful ideas, which in turn, leads to a low level of innovative performance (Rosing and Zacher, 2017).

Combined the above analysis of the “exploration-exploitation” paradox, there is a competitive relationship between explorative and exploitative activities in the aspect of resources, including limited financial and temporal resources (Martin et al., 2019; Pertusa-Ortega et al., 2021). Individuals with a paradox mindset are more likely to experience and embrace the tensions between exploration and exploitation, take advantage of the tensions to simultaneously explore new capabilities, and exploit their accumulated competencies, which leads to high individual ambidexterity. In turn, this is conducive to enhancing employees’ innovative performance. Therefore, we propose the following hypothesis:

***Hypothesis 5*** *: Individual ambidexterity mediates the relationship between employees’ paradox mindset and innovative performance.*

### The chain-mediating effect of role breadth self-efficacy and individual ambidexterity

Innovation is a process of sensing problems, making guesses, building hypotheses, discussing with others, and contradicting habitual actions or “what is desired” (Ford, 1996; Drazin et al., 1999). Sensemaking together with motivation, knowledge, and ability are important factors that determine employees engaged in creative rather than habitual actions (Ford, 1996; Unsworth and Clegg, 2010). We focus on motivation and ability components in Ford’s model and choose capability beliefs and behavioral abilities as the mediating mechanisms to explain why individuals with a paradox mindset undertake innovative action. In terms of how actions happen, one’s expectations of ability influence actions before ability. Furthermore, in line with the findings of Kauppila and Tempelaar (2016) that employees’ general self-efficacy positively predicted individual ambidexterity, we propose that RBSE has a positive effect on individual ambidexterity.

Ambidexterity is difficult to achieve at the individual level (Kauppila and Tempelaar, 2016; Mom et al., 2019). The social cognitive theory emphasizes that self-efficacy belief plays an important role in pursuing complex and difficult goals (Judge et al., 2003; Bledow and Frese, 2009). When individuals perceive that they can engage in explorative and exploitative activities, they will have a great willingness to carry out both activities simultaneously. Specifically, RBSE is more related to these multi-role tasks, dealing with complex and conflicting situations (Phillips and Gully, 1997) and undertaking a wide range of different work behaviors (Parker, 1998).

First, employees with high RBSE believe that they can perform a series of broad roles beyond the formal job description, which motivates them to explore new work roles and tasks (Parker et al., 2006). The more confidence the employees have in fulfilling roles in various domains, the more likely they transfer insights from one domain to another domain (Axtell and Parker, 2003), along with improving their ability to integrate exploration and exploitation across different domains. Moreover, employees with high RBSE prefer conflicting activities (Batt, 2002), and they would like to try to identify new connections between contradictory elements, put forward integrated solutions that emphasize exploration and exploitation as mutually related and complementary, and help them perform both two activities effectively (Smith, 2014).

Second, employees with high RBSE are more confident and proactive to search for novel ideas and alternate among opposing tasks, goals, and thoughts (Phillips and Gully, 1997), which helps them to switch quickly and flexibly between exploitative and explorative activities (Laureiro-Martínez et al., 2015). They are familiar with a range of different roles, so they will be confident in which activities are more suitable for different situations (Bledow et al., 2009). Employees with high RBSE are also more likely to develop a comprehensive understanding of individual ambidexterity and enhance the ability to shift between explorative and exploitative activities, avoiding the trap of only engaging in exploration and exploitation.

To conclude, individuals with a paradox mindset feel confident that they can be proactive and perform a broader role beyond formal job duties, which enhances their behavioral ability to engage in both explorative and exploitative activities and, in turn, promote their innovative performance. Thus, we propose the following hypothesis:

***Hypothesis 6:** Role breadth self-efficacy and individual ambidexterity paly a chain-mediating role in the process of employees’ paradox mindset influencing innovative performance.*

## Materials and methods

### Sample and data collection

We collected data from several enterprises in Beijing, Shanghai, Guangdong, and Jiangsu Province in China, involving IT, machine manufacturing, real estate, and the financial industry. We adopted two ways to collect data. One way is to distribute and receive employees’ questionnaires sealed in envelopes on site. The other way is to send the questionnaires to participants and request them to return the completed questionnaires to investigators *via* e-mail directly. At first, we communicated with the head of the human resource management department in these organizations in advance and selected six trained research team members who work with them to get the list of supervisors and subordinates who voluntarily participated in our study. We used alphabetic and numeric codes (family name and the last four digits of their phone numbers) to match the employees’ questionnaires with their supervisors’ evaluations. Then, we conducted a survey in three ways. At Time 1, we invited 597 employees to participate in our survey, including questions on demographic variables, paradox mindset, and RBSE. After 4 weeks (at Time 2), 562 employees continued to rate their exploration and exploitation. At Time 3 (4 weeks later), the corresponding supervisors evaluated their own demographic information and their subordinates’ innovative performance. A total of 597 employees and 123 supervisors responded to this study. Some responses were excluded because of missing data in the employees’ questionnaires or because some supervisors did not rate their subordinate’s innovative performance. Finally, we analyzed a sample of 480 employees (80.40%) paired with 100 supervisors (81.30%).

In the final sample, the employees had a mean age of 29.88 years (*SD* = 4.58) and an average of 3.52 years’ organization tenure (*SD* = 3.64); 54.40% were women; 70% obtained a bachelor’s degree. The supervisors were 34.06 years old (*SD* = 4.52) on average, they had an average of 5.82 years’ organization tenure (*SD* = 3.66) and an average of 4.80 subordinates; 42.90% were women, and 78.9% obtained a bachelor’s degree.

### Measures

We conducted all measures in Chinese by developing from original English measures with the translation-back-translation procedure (Brislin, 1986). Unless otherwise indicated, responses to all items were on a 5-point Likert-type scale with anchors ranging from 1 (strongly disagree) to 5 (strongly agree).

#### Paradox mindset

We used the 9-item scale from Miron-Spektor et al. (2018) to measure the paradox mindset of employees (α = 0.87). This scale showed good validity in the Chinese sample (Liu et al., 2020). A sample item is “I am comfortable dealing with conflicting demands at the same time.”

#### Role breadth self-efficacy

We used a 7-item scale from Parker (1998) to measure RBSE from employees (α = 0.89). A representative item is “Making suggestions to management about ways to improve the working of your section.”

#### Individual ambidexterity

We used the 11-item scale from Mom et al. (2007) to capture individual ambidexterity, including five items to measure exploration (α = 0.88) and six items to measure exploitation from employees (α = 0.74). We followed the prior studies (Mom et al., 2009; Tempelaar and Rosenkranz, 2019) and argued that individual ambidexterity was indicated by the product of exploration and exploitation. Example items are as follows: To what extent did you engage in work-related activities that can be characterized as follows “Searching for new possibilities with respect to products/services, processes, or markets” and “Activities of which a lot of experience has been accumulated by yourself.” These items used a 7-point Likert-type scale ranging from 1 (never) to 7 (always).

#### Innovative performance

We used a 13-item scale developed by Zhou and George (2001) to measure innovative performance from employees (α = 0.93). A representative item is “Come up with new and practical ideas to improve performance.”

#### Control variables

To rule out alternative explanations, we controlled for employees’ demographic characteristics, including gender, age, education, tenure, and team function. In addition, we also controlled for employees’ role overload and openness to experience. Individuals with high role overload percept that they have inadequate resources to deal with role demands and elicit stress or distraction (Kahn et al., 1964), and then, they are more likely to experience high tensions. Some studies show that role overload has an attenuating effect on the relationship between self-efficacy and goal level to work performance (Brown et al., 2005), and it has a mixed effect on extra-role performance (Huang et al., 2021). According to these studies, we used a 5-item subscale from Peterson et al. (1995) to measure role overload from employees. A sample item is “I feel certain about how much authority I have.” The Cronbach’s α was 0.75. In addition, individuals who are high in openness to experience have a wide range of interests and tend to be open-minded, non-traditional, imaginative, and creative (Costa and McCrae, 1992). They are inclined to be curious about new things and be open to new opinions or ideas (Kaufman, 2013). Previous studies have demonstrated that openness to experience is not only positively related to individual innovation (Park et al., 2018) but also plays a moderating role between experienced creative pressure and creativity (Baer and Oldham, 2006). So, we controlled openness to experience and measured it with a 12-item subscale from the NEO Five-Factor Inventory (Costa and McCrae, 1992). A representative item is “I often enjoy playing with theories or abstract ideas.” Cronbach’s α was found to be 0.56.

### Analytical strategy

First, we performed reliability analysis and confirmatory factor analysis (CFA) to examine the distinctive validity of the variables in this study. Second, we performed descriptive analysis and correlation analysis to provide preliminary support. Third, hierarchical regression was usually implemented to examine whether the effect of variables explained a statistically significant amount of variance in the dependent variable while controlling for the effects of the others, which was often used to manifest the mediating effect (Lankau and Scandura, 2002; Xing and Li, 2022). Therefore, we adopted hierarchical regression analysis to verify the research hypotheses.

Given that our measurement potentially violates independent assumption (one supervisor estimated an average of 4.8 employees), we estimated a fully unconditional model for employees’ innovative performance within and between groups to examine the nested effect of the data. The result showed that the intraclass correlation coefficient (ICC) was 0.043 (less than 0.059), indicating that there was no significant cluster effect for the outcome variable (Cohen, 1988). Furthermore, we calculated two kinds of indicators, including collinearity tolerance (CT) and variance inflation factor (VIF) to evaluate the potential issue of multicollinearity. The CT value for independent variables and control variables was distributed between 0.66 and 0.98, which was greater than 0.10. The VIF value for these variables was distributed between 1.02 and 1.70, which was less than 10. The results indicate that there is no significant multicollinearity among independent variables. Therefore, based on the above analysis, it is appropriate to employ hierarchical regression to verify proposed hypotheses 1–5 in this study. Finally, we also used SPSS macro to examine the indirect effect of employees’ paradox mindset on innovative performance through RBSE and individual ambidexterity.

## Results

### Preliminary analysis

#### The reliability and validity tests

As shown in Table 1, we calculated Cronbach’s alpha, composite reliability (CR), and average variance extracted (AVE) to manifest the reliability of five measures. Cronbach’s alpha of all the measures ranged from 0.74 to 0.93, which was greater than the acceptable level of 0.70. The composite reliability values of focal variables ranged from 0.83 to 0.92, indicating that the measurement items had a high level of internal reliability (Fornell and Larcker, 1981). The average variance extracted denoted the amount of variance in the indicators that was explained by the latent constructs, most of which in this study were acceptable according to Fornell and Larcker (1981). The value of Kaiser–Meyer–Olkin (KMO) of all measures ranged from 0.74 to 0.95, surpassing the acceptable level of 0.70. This indicated that the focal variables were suitable to conduct CFA. All of the factor loadings for the current measurement model were significant in the predicted directions.

**TABLE 1:** Measurement items, reliability, and validity tests.

Constructs	Items	FL	α	CR	KMO	AVE
Paradox mindset	1. When I consider conflicting perspectives, I gain a better understanding of an issue.	0.53	0.87	0.87	0.87	0.44
	2. I am comfortable dealing with conflicting demands at the same time.	0.75				
	3. Accepting contradictions is essential for my success.	0.64				
	4. Tension between ideas energizes me.	0.54				
	5. I enjoy it when I manage to pursue contradictory goals.	0.78				
	6. I often experience myself as simultaneously embracing conflicting demands.	0.56				
	7. I am comfortable working on tasks that contradict each other.	0.76				
	8. I feel uplifted when I realize that two opposites can be true.	0.67				
	9. I feel energized when I manage to address contradictory issues.	0.70				
In your daily work, how confident would you feel?						
Role breadth self-efficacy	1. Analyzing a long-term problem to find a solution.	0.74	0.89	0.83	0.90	0.43
	2. Designing new procedures for your work area.	0.76				
	3. Making suggestions to management about ways to improve the working of your section.	0.70				
	4. Contacting people outside the company (e.g., suppliers, customers) to discuss problems.	0.55				
	5. Helping to set targets/goals in your work area.	0.70				
	6. Representing your work area in meetings with senior management.	0.51				
	7. Visiting people from other departments to suggest doing things differently.	0.60				
To what extent did you, last year, engage in work related activities that can be characterized as follows:						
Exploration	1. Searching for new possibilities with respect to products/services, processes or markets.	0.80	0.88	0.88	0.74	0.60
	2. Evaluating diverse options with respect to products/services, processes or markets.	0.82				
	3. Focusing on strong renewal of products/services or processes.	0.84				
	4. Activities requiring quite some adaptability of you.	0.75				
	5. Activities requiring you to learn new skills or knowledge.	0.63				
Exploitation	1. Activities of which a lot of experience has been accumulated by yourself.	0.63	0.74	0.91	0.74	0.64
	2. Activities which serve existing (internal) customers with existing services/products.	0.67				
	3. Activities of which it is clear to you how to conduct them.	0.80				
	4. Activities primarily focused on achieving short-term goals.	0.80				
	5. Activities which you can properly conduct by using your present knowledge.	0.85				
	6. Activities which clearly fit into existing company policy.	0.88				
Innovative performance	1. He/She suggests new ways to achieve goals or objectives.	0.71	0.93	0.92	0.95	0.48
	2. He/She comes up with new and practical ideas to improve performance.	0.71				
	3. He/She searches out new technologies, processes, techniques, and/or ideas.	0.74				
	4. He/She suggests new ways to increase quality.	0.70				
	5. He/She is a good source of creative ideas.	0.69				
	6. He/She is not afraid to take risks.	0.62				
	7. He/She promotes and champions ideas to others.	0.62				
	8. He/She exhibits creativity on the job when given the opportunity to.	0.64				
	9. He/She develops adequate plans and schedules for the implementation of new ideas.	0.64				
	10. He/She often has new and innovative ideas.	0.71				
	11. He/She comes up with creative solutions to problems.	0.72				
	12. He/She often has a fresh approach to problems.	0.78				
	13. He/She suggests new ways of performing work tasks.	0.76				

FL, factor loading; α, Cronbach’s alpha; CR, composite reliability; AVE, average variance extracted.

We conducted CFA using Mplus 8 to examine the measurement model specifying five separate factors, including employees’ paradox mindset, RBSE, exploration, exploitation, and innovative performance. Due to the sample size relative to the measurement items, we created three parcels for the paradox mindset and six parcels for the innovative behavior with a parceling procedure (Aryee et al., 2007). As shown in Table 2, comparing this model with other alternative ones, the hypothesized five-factors model had the best fit (*χ^2^* = 985.54, *df* = 306, *χ^2^/df* = 3.22, CFI = 0.92, TLI = 0.91, RMSEA = 0.07, and SRMSR = 0.09). This indicated that the study variables are five separate constructs.

**TABLE 2:** Results of the confirmatory factor analysis for the study variables.

Model	*χ^2^*	*df*	Δχ^2^ (Δ *df*)	CFI	TLI	RMSEA	SRMR
Six-factor model: PM; RBSE; EOR; EIT; IP; CMV	888.05	287	−	0.93	0.91	0.07	0.05
Five-factor model: PM; RBSE; EOR; EIT; IP	985.54	306	97.49 (19)	0.92	0.91	0.07	0.09
Four-factor model: PM + RBSE; EOR; EIT; IP	2145.34	318	1159.80 (12)	0.78	0.76	0.11	0.10
Three-factor model: PM + RBSE; EOR + EIT; IP	3089.31	321	943.97 (3)	0.67	0.64	0.13	0.12
Two-factor model: PM + RBSE + EOR + EIT; IP	4143.15	323	1053.84 (2)	0.54	0.50	0.16	0.14
One-factor model: PM + RBSE + EOR + EIT + IP	5078.68	324	935.53 (1)	0.43	0.38	0.18	0.15

*N* = 480. χ^2^, chi-square; *df*, degrees of freedom; CFI, comparative fit index; TLI, Tucker-Lewis index; RMSEA, root mean square error of approximation; SRMR, standardized root mean square residual; PM, paradox mindset; RBSE, role breadth self-efficacy; EOR, exploration; EIT, exploitation; IP, innovative performance; CMV, common method variance.

Although we collected data from employees and their corresponding supervisors to reduce the common method biases effectively, it was still necessary to conduct a common method bias test. We adopted Harman’s single-factor test by loading all of the variables into an exploratory factor analysis to address the issue of common method variance (Podsakoff et al., 2003, p. 889). The unrotated factor solution demonstrated that the variation of the first principal component was 27.72%, which was less than the 50% recommended by Harrison et al. (1996) and did not account for half of the total variation (64.41%) based on the eigenvalues greater than 1. Furthermore, we also adopted the unmeasured latent method factor technique to minimize the detrimental effects of method biases (Podsakoff et al., 2012, p. 553). We added common method variance (CMV) into CFA and compared the changes in the model parameter index to test the influence of common method variance. As shown in Table 2, as compared to the five-factor model, there was no significant change in the indexes of the six-factor model (*χ^2^* = 888.05, *df* = 287, *χ^2^/df* = 3.09, CFI = 0.93, TLI = 0.91, RMSEA = 0.07, and SRMR = 0.05). The above analysis demonstrated that the common biases in this study were not serious.

#### Descriptive analysis

The means, standard deviations, reliability coefficients, and correlations among variables in this study are shown in Table 3. Employees’ paradox mindset is positively related to RBSE (*r* = 0.40, *p* < 0.01), exploitation activity (*r* = 0.16, *p* < 0.01), individual ambidexterity (*r* = 0.16, *p* < 0.01), and innovative performance (*r* = 0.37, *p* < 0.01). Employees’ RBSE correlates positively with exploration activity (*r* = 0.42, *p* < 0.01), exploitation activity (*r* = 0.13, *p* < 0.01), individual ambidexterity (*r* = 0.41, *p* < 0.01), and innovative performance (*r* = 0.59, *p* < 0.01). Employees’ individual ambidexterity is positively correlated with innovative performance (*r* = 0.36, *p* < 0.01).

**TABLE 3:** Descriptive statistics and correlations among the study variables.

Variables	*M*	*SD*	1	2	3	4	5	6	7	8	9	10	11	12
(1) Gender	1.46	0.50	1											
(2) Age	29.88	4.58	0.00	1										
(3) Education	3.70	0.74	0.03	0.01	1									
(4) Tenure	3.52	3.64	0.00	0.55**	−0.28**	1								
(5) Role overload	2.73	0.69	–0.01	0.02	0.01	0.04	1							
(6) Openness to experience	3.41	0.38	–0.09	0.00	–0.05	0.07	−0.11*	1						
(7) Paradox mindset	3.59	0.60	–0.06	0.03	0.00	0.15**	–0.03	0.22**	1					
(8) Role breadth self-efficacy	3.71	0.73	−0.09*	0.18**	0.10*	0.20**	–0.08	0.21**	0.40**	1				
(9) Exploration	5.06	1.06	–0.05	0.13**	–0.07	0.11*	–0.01	0.25**	0.04	0.42**	1			
(10) Exploitation	5.22	0.87	0.06	0.17**	−0.10*	0.12**	–0.07	0.25**	0.16**	0.13**	0.22**	1		
(11) individual ambidexterity	26.58	8.04	–0.00	0.19**	−0.09*	0.15**	–0.05	0.32**	0.16**	0.41**	0.83**	0.71**	1	
(12) Innovative performance	3.76	0.54	0.04	0.14**	0.00	0.19**	–0.09	0.12**	0.37**	0.59**	0.27**	0.21**	0.36**	1

*N* = 480. *p < 0.05, **p < 0.01.

### Hypothesis tests

Given that individual ambidexterity was calculated by the product of exploration and exploitation, there is a difference in magnitude scale between individual ambidexterity and the rest variables in the theoretical model. We converted the product terms into standardized scores to avoid this difference leading to changes in the prediction of independent variables on individual ambidexterity and other variables (Zhang et al., 2022). The results of the hierarchical regression analysis are shown in Table 4. Model 7 presents that after controlling for gender, age, education, tenure, role overload, and openness to experience, employees’ paradox mindset has a positive association with innovative performance (β = 0.31, *p* < 0.01), providing support for hypothesis 1. In Model 2, employees’ paradox mindset positively influences RBSE (β = 0.42, *p* < 0.01), and hypothesis 2 is supported. After controlling for control variables and paradox mindset, Model 8 shows that RBSE has a significant positive influence on innovative performance (β = 0.39, *p* < 0.01), and the regression coefficient of employees’ paradox mindset to innovative performance decreases (β = 0.15, *p* < 0.01), demonstrating that RBSE mediates the relationship between employees’ paradox mindset and innovative performance. Therefore, hypothesis 3 is verified. From Model 4, employees’ paradox mindset positively influences individual ambidexterity (β = 0.62, *p* < 0.01), providing support for hypothesis 4. According to Model 9, after controlling control variables and paradox mindset, individual ambidexterity has a significant positive influence on innovative performance (β = 0.16, *p* < 0.01), and the regression coefficient of paradox mindset on innovative performance decreases (β = 0.21, *p* < 0.01), indicating that individual ambidexterity also mediates the relationship between employees’ paradox mindset and innovative performance. Thus, hypothesis 5 is supported. In Model 10, all study variables enter the equation, and we can observe that the positive effect of a paradox mindset (β = 0.10, *p* < 0.01) and RBSE (β = 0.35, *p* < 0.01) on innovative performance is significantly reduced than the regression coefficient of the paradox mindset (β = 0.31, *p* < 0.01) in the Model 7 and RBSE (β = 0.39, *p* < 0.01) in the Model 8. Individual ambidexterity also has a positive influence on employee innovative performance (β = 0.10, *p* < 0.01). Therefore, hypothesis 6 is verified.

**TABLE 4:** Results of hierarchical regression analysis.

Variables	Role breadth self-efficacy		Individual ambidexterity				Employees’ innovative performance			
			
	Model 1	Model 2	Model 3	Model 4	Model 5	Model 6	Model 7	Model 8	Model 9	Model 10
**Control variables**										
Gender	−0.12 (0.06)	−0.10 (0.06)	−0.05 (0.10)	−0.01 (0.10)	0.03 (0.09)	0.05 (0.05)	0.07 (0.05)	0.11 (0.04)	0.07 (0.04)	0.10** (0.04)
Age	0.01 (0.01)	0.02 (0.01)	0.01 (0.01)	0.02 (0.01)	0.01 (0.01)	0.01 (0.01)	0.01 (0.01)	0.03 (0.01)	0.01 (0.01)	0.02 (0.01)
Education	0.17** (0.05)	0.15** (0.04)	0.12 (0.07)	0.08 (0.07)	0.02 (0.07)	0.04 (0.03)	0.03 (0.03)	−0.03 (0.03)	0.01 (0.03)	−0.04 (0.03)
Tenure	0.04 (0.01)	0.03** (0.01)	0.01 (0.02)	−0.01 (0.02)	−0.02 (0.02)	0.03 (0.01)	0.02 (0.01)	0.01 (0.01)	0.16* (0.01)	0.01 (0.01)
Role overload	−0.07 (0.05)	−0.07 (0.04)	−0.05 (0.07)	−0.05 (0.07)	−0.02 (0.07)	−0.06 (0.04)	−0.06 (0.03)	−0.03 (0.03)	−0.05 (0.03)	−0.03 (0.03)
Openness to experience	0.37** (0.08)	0.23** (0.08)	0.29* (0.13)	0.09 (0.13)	−0.01 (0.12)	0.15* (0.06)	0.05 (0.06)	−0.04 (0.05)	0.04 (0.06)	−0.04 (0.05)
**Independent variable**										
Paradox mindset		0.42** (0.05)		0.62** (0.08)	0.43** (0.07)		0.31** (0.04)	0.15** (0.04)	0.21** (0.04)	0.10** (0.04)
**Mediator**										
Role breadth self-efficacy					0.44** (0.07)			0.39** (0.03)		0.35** (0.03)
Individual ambidexterity									0.16** (0.02)	0.10** (0.02)
*R* ^2^	0.12	0.24	0.03	0.14	0.20	0.06	0.17	0.39	0.26	0.42
*ΔR^2^*	–	0.12	–	0.11	0.06	–	0.11	0.22	0.09	0.03
*F*	10.92**	20.68**	2.02	10.48**	14.91**	5.09	14.13	37.81**	20.74**	37.90**
*ΔF*	–	69.72**	–	59.74**	39.90**	–	64.30**	168.46**	55.51**	23.90**

*N* = 480. *p < 0.05, **p < 0.01. The unstandardized coefficients were reported. The values in the parentheses were standard errors.

**FIGURE 2:**
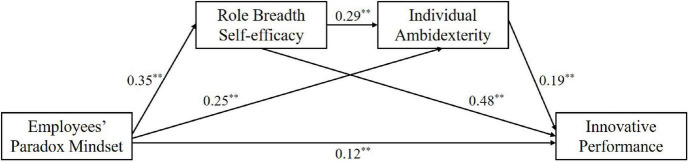
Standardized estimates of the path coefficients.

Based on the above analysis, we also used a more powerful bootstrapping method to examine the robustness of mediation. We conducted this test by PROCESS macro (Hayes, 2017), and the results are shown in Table 5. The total indirect effect of employees’ paradox mindset on innovative performance is 0.23 (*SE* = 0.03, 95% confidence interval [CI] = [0.17, 0.30], excluding zero), accounting for 65.71% of total effects. The indirect effect of a paradox mindset on employees’ innovative performance through RBSE is 0.17 (*SE* = 0.03, 95% confidence interval [CI] = [0.12, 0.23], excluding zero), accounting for 48.57% of total effects. This shows that the mediating effect is significant and hypothesis 3 is further verified. The indirect effect of a paradox mindset on employees’ innovative performance through individual ambidexterity is 0.05 (*SE* = 0.02, 95% confidence interval [CI] = [0.02, 0.08], excluding zero), accounting for 14.29% of total effects, indicating that the mediating effect of individual ambidexterity is significant. Thus, hypothesis 5 is further verified. The indirect effect of a paradox mindset on employees’ innovative performance successively through RBSE and individual ambidexterity is 0.02 (*SE* = 0.01, 95% confidence interval [CI] = [0.01, 0.04], excluding zero), accounting for 5.71% of total effects, indicating that the chain-mediating effect is significant, providing support for hypothesis 6. We validate the chain-mediating role of RBSE and individual ambidexterity in the process of paradox mindset affecting employees’ innovative performance. In addition, we construct a structural equation model to examine the relationship among these study variables, and Figure 2 shows the path coefficients of the structural equation model.

**TABLE 5:** The mediation effect analysis.

Mediation path	Effect	SE	Bootstrap 95% CI	Relative mediation effect
1. Total indirect effect	0.23	0.03	[0.17, 0.30]	65.71%
2. Paradox mindset → Role breadth self-efficacy → Innovative performance	0.17	0.03	[0.12, 0.23]	48.57%
3. Paradox mindset → Individual ambidexterity → Innovative performance	0.05	0.02	[0.02, 0.08]	14.29%
4. Paradox mindset → Role breadth self-efficacy → Individual ambidexterity → Innovative performance	0.02	0.01	[0.01, 0.04]	5.71%

*N* = 480. Model 6 (2 mediators) in PROCESS macro; bootstrap resample = 5,000; SE indicates standard error; CI indicates confidence interval.

## Discussion

The current study provides new insights into how employees’ paradox mindset affects innovative performance from an agentic perspective. This study finds that employees’ paradox mindset has a positive influence on innovative performance through RBSE and individual ambidexterity. In addition, each RBSE and individual ambidexterity plays a chain-mediating role between employees’ paradox mindset and innovative performance. In another words, adopting a paradox mindset can enhance an individual’s capability beliefs and behavioral abilities to encourage employees to engage in innovation.

### Theoretical contributions

From a theoretical perspective, our results contribute in three main ways. First, our results show that the sense of energy gaining from the activation of a paradox mindset improves the individual’s confidence in their ability to undertake expanded roles (involving proactive, interpersonal, and integrative tasks), which promotes their innovative performance. On one hand, the present study develops the paradox mindset as a new trait-like antecedent causing the changes in RBSE, which goes beyond individual characteristics such as self-esteem, proactivity (Parker, 1998; Parker et al., 2006; Chen et al., 2013), and over-qualification (Zhang et al., 2016), contributing to the research on the generation of RBSE. On the other hand, we respond to those calls about the emphasis on the agentic nature of innovative behavior (Ng and Lucianetti, 2016; Rodrigues and Rebelo, 2021). This implies that employees with a paradox mindset intentionally make innovative things happen through their own actions. In line with previous studies, the adoption of a paradox mindset can bring creative values (Miron-Spektor et al., 2011b; Leung et al., 2018). However, we can understand the positive influence of a paradox mindset on innovative performance based on personal agency, which also provides new insights into the literature on the effect of the paradox mindset.

Second, our study provides empirical evidence for managing the tensions of exploration and exploitation from the lens of paradox theory by linking employees’ paradox mindset with individual ambidexterity, which follows the calls for realizing individual ambidexterity through a paradox lens (Papachroni et al., 2015; Papachroni and Heracleous, 2020). Typically, there are two viewpoints on the understanding of ambidexterity. Some scholars regard exploration and exploitation as two ends of the same continuum (March, 1991), competing for limited resources and trying to find the balance between the two to manage the tensions between these activities. Other scholars view explorative and exploitative activities as orthogonal and independent from each other. Furthermore, organizations can realize ambidexterity by separating spatially or temporally and maintaining a high level of both these two activities (Gupta et al., 2006; Lubatkin et al., 2006). However, a paradox perspective goes beyond the above two approaches to ambidexterity, which implies that adopting a paradox mindset enables individuals to develop the behavioral capacity to maintain and balance a high level of both exploration and exploitation, in turn enhancing innovative performance. That is to say, the paradoxical management of ambidexterity tensions moves beyond the separation thesis toward synthesis or transcendence of competing activities (Papachroni et al., 2015; Martin et al., 2019). Some studies find that the individuals’ paradoxical cognition is helpful in facilitating senior managers to handle the conflicts of explorative and exploitative innovation (Smith and Tushman, 2005), including that two activities can be reinforced between each other and promote innovation (Andriopoulos and Lewis, 2009; Zacher et al., 2016).

Third, the current study expands our understanding of how certain factors may influence and determine individuals’ engagement in innovation. Given the paradoxical nature of innovation, our findings suggest that RBSE and individual ambidexterity sequentially play the chain-mediating role between paradox mindset and innovative performance, which provides a possible interpretation of how employees manage these competing goals (Madjar et al., 2011). Ford (1996) argues that undertaking creative actions is a deliberate process and is regarded as an alternative to the competing option of habitual actions, involving the combined influence of sensemaking, motivation, knowledge, and ability. Overall, the chain effect of RBSE and individual ambidexterity confirms that capability beliefs and behavioral abilities sequentially drive employees to engage in innovative behaviors. In addition, this study advances our knowledge of the psychological mechanisms regarding how individuals with a paradox mindset respond to innovation paradox, making innovative things happen, which verifies that a paradox lens can bring new insights and creative benefits to organizational actors (Miron-Spektor and Erez, 2017; Miron-Spektor et al., 2018).

### Limitations and research directions

This study is subjected to potential limitations, which brings about some opportunities for future research. First, we only utilize a cross-sectional research design with data collected at three-time points to examine the relationships among focal variables in the aforementioned research model. However, recent studies have paid more attention to the relationships between individual characteristics and increases in innovative behavior (Ng and Lucianetti, 2016), or creativity trajectories, that is, individuals can improve and sustain their innovation over time (Miron-Spektor et al., 2022). With regard to collecting repeated measures from the same individual over time and examining the mediating effect from a change perspective is a more rigorous approach to verifying theoretical models (Pitariu and Ployhart, 2010; Ployhart and Vandenberg, 2010). Based on this, future studies can use a longitudinal research design to capture a dynamic mediated relationship among individuals’ paradox mindset and innovative performance from a within-individual change perspective to advance our knowledge on the changes in individual innovation over time.

Second, although we examine the mediating process about how a paradox mindset as a personal trait inspires individual innovation, there is a lack of attention to the boundary conditions of whether the influence of a paradox mindset is contingent on the effect of the situation in which individuals operate. Recently, Knippenberg and Hirst (2020) propose the motivational lens model of person-in-situation creativity, which regards individuals as active agents filtering, interpreting, and dealing with the situation (Barrick et al., 2013). Moreover, different traits enable individuals response to different aspects of the situation. We propose that a paradox mindset is an approach to the paradoxical tensions caused by explorative and exploitative activities; however, future research should focus on exploring the factors responsible for the activation of the positive effect of a paradox mindset from an interactionist perspective, such as paradoxical leadership (Zhang et al., 2015; Waldman and Bowen, 2016) and high-involvement HR practices (Combs et al., 2006), which would describe an integrated and comprehensive view on how and when adopting a paradox mindset results in creative values.

Third, drawing on the tenets of social cognitive theory and innovation paradox, we examine the mediating roles of RBSE and individual ambidexterity between employees’ paradox mindset and innovative performance, which go beyond the mediating role of thriving at work from a motivational perspective (Liu et al., 2020). In addition, based on the job demands-resources model (Bakker and Demerouti, 2017), Yin (2021) finds that a paradox mindset has a positive influence on work engagement through seeking challenges and individual unlearning, which also inspires us to think about when confronted with the challenging situation with competing demands, individuals with a paradox mindset may be more creative *via* the mediated effect of self-set goal level, feedback seeking, learning goal orientation, etc. (Keating and Heslin, 2015). Furthermore, it is interesting that future research examines whether individual unlearning (Navarro and Moya, 2005; Hislop et al., 2014) plays the mediating role between a paradox mindset and innovation.

### Practical implications

Our results have certain managerial implications. First, the results imply that managers should understand the agentic nature of innovative behaviors if they want to motivate their subordinates to undertake innovating roles. Ng and Lucianetti (2016) find that creative, persuasion, and change self-efficacy are positively related to the innovative process; however, we find that RBSE captures individuals’ cognitive beliefs on carrying out a series of expanded roles, which can meet the frequent and ongoing change and demands for improvement in modern organizations. This suggests that creativity training should not be restricted to domain-specific self-efficacy but should focus on enhancing proactive motivation states to foster individual innovation as well as yield a high return on human resource investment. For example, based on the viewpoints of Bandura (1982), managers can also attempt to improve employees’ self-efficacy beliefs by setting up competent work tasks and providing examples of successful experiences.

Second, our findings suggest that increases in a paradox mindset are related to the increases in RBSE and suggest to managers that the energy from adopting a paradox mindset is helpful to increase employees’ confidence in undertaking innovative behaviors. Furthermore, the results show that the individual characteristics of organizational actors have a significant influence on their behavioral capability to assume innovative work roles. Thus, for people management, the managers should seek to recruit employees with a high paradox mindset to promote individual innovation in the workplace, in turn improving the sustainable development of the organization. In addition, organizations also can help employees to develop a paradox mindset by training them to deal with conflicting agendas through a paradox lens (Knight and Paroutis, 2017).

Third, our results indicate that RBSE (capability beliefs) and individual ambidexterity (behavioral abilities) are the important factors facilitating creative actions. Thus, organizations should put more emphasis on the relationships between individual ambidexterity and innovation. In management practices, on the one hand, given that ambidexterity is a key individual competence in most jobs (Kauppila and Tempelaar, 2016), managers might take measures to cultivate this behavioral capability by shaping employees’ self-efficacy beliefs on undertaking broader work roles beyond the present job duties. On the other hand, organizations that aim to foster employees’ innovation could encourage engagement in both high explorative and exploitative activities by linking ambidextrous goals to tangible or intangible incentives.

## Conclusion

Drawing on the tenets of social cognitive theory and innovation paradox, the present study demonstrates that the influence of a paradox mindset on employees’ innovative performance can be explained by RBSE and individual ambidexterity as two different underlying mechanisms. Specifically, when confronted with an “exploration-exploitation” paradoxical situation, employees who adopt a paradox mindset not only hold confidence to fulfill a broad role, including undertaking certain broader and proactive tasks, but also feel energized to engage in both explorative and exploitative activities and then foster individual innovation. Furthermore, the more employees feel confident in undertaking expanded roles, the higher they develop the behavioral capability to respond to the tensions caused by exploration and exploitation. In another words, employees with a paradox mindset become motivated to engage in innovation through their self-endeavors.

Our findings provide empirical support that employees adopting a paradox mindset are more confident and competent to balance rather than separate them. Employees can transfer individual ambidexterity from a view of dualism between exploration and exploitation to an assumption of dynamic polarities, which may drive them to shift between two activities constantly to achieve the dynamic equilibrium (Smith and Lewis, 2011). In practice, managers might perform the policy of human resource management to promote the creative value of a paradox mindset, such as recruiting employees with a high level of paradox mindset, launching personnel training to develop employees’ paradox mindset, and further enhancing their confidence in performing broader tasks and their ability to manage ambidexterity tensions. Besides, organizations also could create a supportive culture and set a good example to cultivate their subordinates’ RBSE, improving individual ambidexterity to engage in innovation.

## Data availability statement

The raw data supporting the conclusions of this article will be made available by the authors, without undue reservation.

## Ethics statement

Ethical review and approval was not required for the study on human participants in accordance with the local legislation and institutional requirements. The patients/participants provided their written informed consent to participate in this study.

## Author contributions

YL contributed to the research ideas, collected the data, run the data, and wrote the full manuscript. HZ helped in theory building and revised the manuscript. Both authors made substantial contribution to the article and approved the submitted version.
